# Shared alterations in hippocampal structural covariance in subjective cognitive decline and migraine

**DOI:** 10.3389/fnagi.2023.1191991

**Published:** 2023-06-20

**Authors:** Chia-Lin Tsai, Kun-Hsien Chou, Pei-Lin Lee, Chih-Sung Liang, Chen-Yuan Kuo, Guan-Yu Lin, Yu-Kai Lin, Yi-Chih Hsu, Chien-An Ko, Fu-Chi Yang, Ching-Po Lin

**Affiliations:** ^1^Department of Neurology, Tri-Service General Hospital, National Defense Medical Center, Taipei City, Taiwan; ^2^Brain Research Center, National Yang Ming Chiao Tung University, Taipei City, Taiwan; ^3^Institute of Neuroscience, National Yang Ming Chiao Tung University, Taipei City, Taiwan; ^4^Department of Psychiatry, Beitou Branch, Tri-Service General Hospital, National Defense Medical Center, Taipei City, Taiwan; ^5^Aging and Health Research Center, National Yang Ming Chiao Tung University, Taipei City, Taiwan; ^6^Graduate Institute of Medical Sciences, National Defense Medical Center, Taipei City, Taiwan; ^7^Department of Radiology, Tri-Service General Hospital, National Defense Medical Center, Taipei City, Taiwan; ^8^Department of Biomedical Imaging and Radiological Sciences, National Yang Ming Chiao Tung University, Taipei City, Taiwan

**Keywords:** subjective cognitive decline, network, gray matter volume, migraine, hippocampus, structural covariance (SC)

## Abstract

**Introduction:**

Subjective cognitive decline (SCD) and migraine are often comorbid. Hippocampal structural abnormalities have been observed in individuals with both SCD and migraine. Given the known structural and functional heterogeneity along the long axis (anterior to posterior) of the hippocampus, we aimed to identify altered patterns of structural covariance within hippocampal subdivisions associated with SCD and migraine comorbidities.

**Methods:**

A seed-based structural covariance network analysis was applied to examine large-scale anatomical network changes of the anterior and posterior hippocampus in individuals with SCD, migraine and healthy controls. Conjunction analyses were used to identify shared network-level alterations in the hippocampal subdivisions in individuals with both SCD and migraine.

**Results:**

Altered structural covariance integrity of the anterior and posterior hippocampus was observed in the temporal, frontal, occipital, cingulate, precentral, and postcentral areas in individuals with SCD and migraine compared with healthy controls. Conjunction analysis revealed that, in both SCD and migraine, altered structural covariance integrity was shared between the anterior hippocampus and inferior temporal gyri and between the posterior hippocampus and precentral gyrus. Additionally, the structural covariance integrity of the posterior hippocampus-cerebellum axis was associated with the duration of SCD.

**Conclusion:**

This study highlighted the specific role of hippocampal subdivisions and specific structural covariance alterations within these subdivisions in the pathophysiology of SCD and migraine. These network-level changes in structural covariance may serve as potential imaging signatures for individuals who have both SCD and migraine.

## 1. Introduction

Subjective cognitive decline (SCD) is a self-reported worsening of memory or more frequent memory complaints despite normal performance on objective neuropsychological tests (Jessen et al., 2014), with a prevalence of approximately 10.4–18.8% in the United States (Taylor et al., 2018). SCD is associated with a higher risk of progression to cognitive impairment and conversion to dementia in adults. Although SCD may be a potential early indicator of cognitive impairment and is a topic of considerable research interest (van Oijen et al., 2007), its pathophysiology remains largely unknown. Accumulating evidence suggests that SCD is associated with neuropsychiatric and medical disorders, such as migraine (Lee et al., 2017; Chu et al., 2020).

Migraine is characterized by intermittent attacks of pulsating, unilateral, and moderate to severe headaches associated with physiological and emotional stressors (Borsook et al., 2012). Approximately 10–20% of the global population experience migraines, which significantly impact daily life and cause substantial functional impairments (Vos et al., 2016). Recurrent headaches and poor memory or cognitive decline are common complaints (Schwedt, 2013; Santangelo et al., 2016). During postictal periods, migraineurs reportedly have poorer psychomotor speed, attention, and verbal memory performance than non-migraineurs (O’Bryant et al., 2005). Higher migraine frequency correlates with higher symptom scores for subjective memory complaints, particularly among patients with aura (Chu et al., 2020).

The hippocampus exerts negative feedback on the hypothalamic–pituitary–adrenal axis. This region is influenced by stress and glucocorticoids, which act in concert with excitatory amino acids and other extracellular and intracellular mediators. Elevated levels of these mediators and their activation under chronic stress may change the structure and function of the hippocampus (Rothman and Mattson, 2010). Since migraine attacks are repeated stressors, alterations in hippocampal structure and function may significantly contribute to migraine pathophysiology. Indeed, migraineurs have lower hippocampal volume and stronger hippocampal-cortico-limbic connectivity than healthy controls (Maleki et al., 2013). The hippocampus also plays a pivotal role in memory processing. Previous structural magnetic resonance imaging (MRI) studies have shown that individuals with SCD have decreased gray matter (GM) volume (GMV) in the hippocampus and entorhinal cortex (Liang et al., 2020). Together, these findings suggest that hippocampal alterations may co-exist in both SCD and migraine.

There is a growing body of work investigating anatomical and functional long-axis (anterior-to-posterior) hippocampal variations. Anterior hippocampal connections to the cortical and subcortical areas differ significantly from posterior hippocampal connections. A resting-state functional MRI study provided evidence that the anterior hippocampus communicates with the amygdala, hypothalamus, and anterolateral temporal lobes, whereas the posterior hippocampus communicates with the cuneus, precuneus, anterior and posterior cingulate cortex, inferior parietal cortex, and parts of the thalamus (Poppenk and Moscovitch, 2011). However, recent evidence from animal studies disclosed cognitive and affective specializations within the anterior and posterior hippocampus, respectively (Fanselow and Dong, 2010). Moreover, Gilboa et al. (2004) demonstrated a long-axis interaction of memory remoteness in a cued autobiographical recall task, with recent memories clustering in the anterior hippocampus. Therefore, understanding detailed functional and structural alterations of the anterior and posterior hippocampal subregions and corresponding brain connections might provide insights into neurocognitive mechanisms underlying migraine and SCD.

To recognize the impact of disease on regional morphological characteristics of associated brain areas, large-scale structural covariance (SC) network (SCN) analysis has been recently proposed for identifying inter-regional coordination between different anatomical brain areas (Spreng et al., 1991; Chou et al., 2015). Meanwhile, recent studies have indicated that inter-regional coordination configurations between the cerebellum and remote cortical regions are related to migraine prognosis (Liu et al., 2020). Thus, comparisons of regional morphological brain features and a global large-scale SCN analysis could offer two distinct but complementary methods for exploring anatomical brain changes and further uncovering the similar pathophysiology between the two disorders. Accordingly, this study investigated shared patterns of neuroanatomical alterations in the hippocampal subregions associated with SCD and migraine using a seed-based large-scale SCN analysis and network-level conjunction analyses.

## 2. Materials and methods

### 2.1. Patient population

We consecutively enrolled individuals with SCD or migraine at the outpatient neurological clinic of the Tri-Service General Hospital, Taiwan. Healthy controls were recruited from the Taipei community by advertisement. Before baseline MRI scans, all participants (including individuals with SCD or migraine and healthy controls) underwent standardized clinical evaluations, including medical history interviews, neurologic examinations, and a battery of neuropsychological tests. Written informed consent was obtained from each participant before the study. The Institutional Review Board of the Tri-Service General Hospital approved the study protocol.

Migraine was diagnosed according to the third edition of the International Classification of Headache Disorders (Headache Classification Committee of the International Headache Society [IHS], 2013). All migraineurs completed a structured questionnaire on demographics and headache profiles during their first visit. They kept a headache diary after recruitment. The Migraine Disability Assessment Questionnaire (MIDAS), visual analog scale (VAS), and Headache Impact Test-6 were used to assess migraine-related disability (Stewart et al., 2001), subjective perception of average pain intensity (Wewers and Lowe, 1990), and headache impact (Kosinski et al., 2003), respectively.

Diagnosis and inclusion criteria for SCD were based on accepted research criteria (Jessen et al., 2014): (a) self-reported experience of persistent memory decline compared to the past 5 years, which was further confirmed by informants; (b) performance within the normal range on the Mini-Mental State Examination and the Montreal Cognitive Assessment (adjusted for age, sex, and education); and (c) a score of 0 on the Clinical Dementia Rating. Furthermore, we used a 24-item SCD questionnaire (SCD-Q) to assess memory (11 items), language (6 items), and executive function (7 items). Each question response was restricted to “yes/no” based on the perceived decline in each domain, and the total score ranged from 0 to 24, with higher scores indicating a greater subjective perception of cognitive decline over the past 2 years (Rami et al., 2014). Other demographic and clinical data, including sex, age, education years, Beck’s Depression Inventory (BDI) score (Beck et al., 1961), Insomnia Severity Index (ISI) score (Morin et al., 2011), duration of SCD, and migraine frequency and duration, were also evaluated.

The control group included age-, sex-, and handedness-matched volunteers without cognitive decline concerns or migraine and with normal neuropsychological test scores.

Exclusion criteria for participants were as follows: (1) diagnosis of primary headache disorder other than migraine; (2) age < 18 or >65 years; (3) hypertension, diabetes, cardiac diseases, or respiratory diseases; (4) history of cerebrovascular disease; (5) other neurological (neurodegenerative diseases, epilepsy, or head injury) or psychiatric (insomnia, psychosis, or depression) diseases; (6) alcohol or illicit drug abuse or current psychoactive medication intake; (7) structural lesion(s) on brain MRI; (8) MRI contraindications; or (9) migraine patients who reported subjective memory complaints.

### 2.2. Neuroimaging

All anatomical scans were acquired using a 3.0T Discovery MR750 scanner (General Electric Healthcare, Milwaukee, WI, USA) with an eight-channel head array coil. T1-weighted scans were acquired with a three-dimensional inversion recovery prepared fast spoiled gradient recalled sequence with the following parameters: repetition time/echo time/inversion time = 10.17/4.16/450 ms, flip angle = 12°, number of excitations = 1, field of view = 256 × 256 mm^2^, matrix size = 256 × 256, 172 slices, and voxel size = 1 × 1 × 1 mm^3^ (without any inter-slice gap and interpolation). Before further image processing procedures, an experienced neuroradiologist examined all scans to exclude individuals with structural abnormalities and substantial head motion.

### 2.3. Preprocessing procedure for brain anatomical MRI

Raw Digital Imaging and Communications in Medicine format files were sorted into individual directories using custom scripts. The sorted files were further converted to the standard NIfTI format using the dcm2niix toolbox,^1^ renamed, and organized into their corresponding subject-specific directories according to brain imaging data structure standards (Gorgolewski et al., 2016). Subsequently, all scans were reoriented to obtain an approximate image origin using a center-of-mass approach. To extract tissue volume maps for the following whole-brain seed-to-voxel SCN analysis, an enhanced voxel-based morphometry analytical pipeline was applied using Statistical Parametric Mapping 12 (SPM12, version 7487; Wellcome Institute of Neurology, University College London, UK) in a MATLAB environment (version R2015b; Mathworks, Natick, MA, USA). Briefly, each participant’s native-space T1-weighted scan was corrected for bias-field inhomogeneities and then segmented into GM, white matter (WM), and cerebrospinal fluid (CSF) with enhanced tissue probability maps (Lorio et al., 2016). This modified, validated segmentation procedure provides better segmentation results for subcortical areas which are the major target regions of interest (ROIs) in the current study. To report all subsequent voxel-wise statistical results achieving a more precise between-subject image alignment, these native-space segmented GM and WM tissue maps were rigidly aligned to the standard Montreal Neurological Institute (MNI) space and warped to the final 1.5-mm isotropic group average tissue templates (generated from all participants) using the geodesic shooting registration algorithm, available in the SPM12 Shoot toolbox (Ashburner and Friston, 2011). These individual MNI-space GM tissue segments were scaled by the number of expansions and contractions to preserve actual tissue volume information before and after spatial normalization. Finally, the MNI-space modulated GM maps were smoothed with an isotropic 8-mm full width at half maximum Gaussian kernel. The global tissue volume and total intracranial volume (TIV = GM+WM+CSF volumes) were also calculated from each individual native-space T1-weighted scan. These global tissue measurements were used to account for individual differences in the overall brain size.

### 2.4. Quality assessment of the MRI dataset

Two additional steps were applied to ensure sufficient image quality for further statistical analyses. First, the MRI quality control tool MRIQC^2^ was used to quantify the degree of head motion in each scan (Esteban et al., 2017). The entropy focus criterion (EFC) index, estimated based on the Shannon entropy of voxel intensities of the T1-weighted scans, was used as an objective index to indicate the degree of head motion. Additionally, a covariance-based sample homogeneity measure was implemented using the computational anatomy toolbox (CAT12)^3^ to evaluate data quality for all MNI-space-modulated GM maps. According to the data homogeneity criteria suggested by this toolbox, no participants were considered potential outliers.

### 2.5. ROI definition: anterior and posterior hippocampus segmentation

**FIGURE 1:**
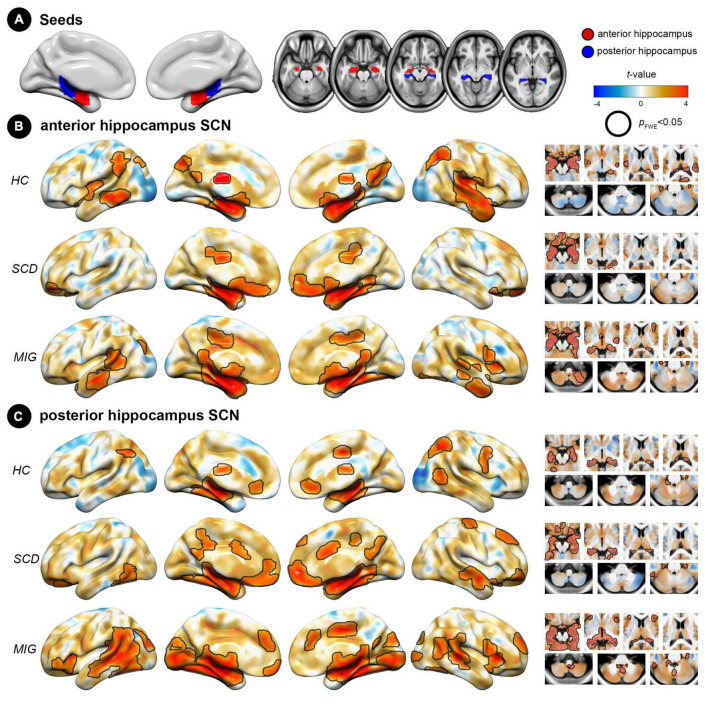
Spatial pattern of hippocampus structural covariance network (SCN) in each group (HC, SCD, and migraine). **(A)** Seed regions for the hippocampus: anterior (red) and posterior (blue) division of the hippocampus. **(B)** Covariance patterns from the anterior hippocampus were mapped in each group. **(C)** Covariance patterns from the posterior hippocampus were mapped in each group. Hot/cold colors indicate the positive/negative correlation to the seed. Black outlines indicate significant regions at a corrected level for threshold *p*-values < 0.05. FWE, family wise error; HC, healthy control; MIG, migraine; SCD, subjective cognitive decline; SCN, structural covariance network.

The hippocampal ROI was identified using the Automated Anatomical Labeling atlas (Tzourio-Mazoyer et al., 2002) of the Wake Forest University PickAtlas toolbox (Maldjian et al., 2003). We further subdivided the hippocampal ROI into anterior and posterior parts by choosing cutoff MNI-space coordinates (Poppenk et al., 2013; Persson et al., 2014). The resulting ROIs ranged along the *y*-axis between −2 and −18 for the anterior and between −24 and −42 for the posterior regions (Figure 1A). Finally, the left and right segments were combined to obtain a single bilateral seed per region. Thus, two hippocampal seed ROIs were generated for the SCN analyses. Subsequently, for each individual MNI-space modulated GM map, the voxels corresponding to the respective region were averaged to represent the regional GM volume information of the seed ROIs.

### 2.6. Data analyses

#### 2.6.1. Demographic data, clinical evaluations, and global tissue volumes

All statistical analyses of demographic variables, clinical evaluations, and global tissue volumes were performed using the Statistical Package for Social Sciences (SPSS, V.20, Armonk, NY, USA). Analysis of variance and Pearson’s chi-square test were used to compare continuous (age, education years, and EFC index) and categorical (sex) data between groups. Moreover, analysis of covariance was performed to compare multiple clinical evaluations and global tissue volumes (GMV, WM volume, CSF volume, TIV, ISI, and total BDI score) between groups with corresponding nuisance variables (Table 1). A *p*-value of <0.05 was considered statistically significant.

**TABLE 1:** Demographics and clinical characteristics of study participants.

Demographic variables	HC	SCD	MIG	*p*-value
	**(*n* = 37)**	**(*n* = 38)**	**(*n* = 48)**	
Age (years)	42.2 ± 11.9	45.7 ± 12.2	40.7 ± 8.7	0.100^a^
Sex (male/female)	14/23	12/26	14/34	0.691^b^
Education years	14.1 ± 2.8	14.4 ± 3.1	15.4 ± 2.1	0.080^a^
GMV	0.699 ± 0.055	0.684 ± 0.070	0.699 ± 0.060	0.876^c^
WMV	0.411 ± 0.053	0.404 ± 0.058	0.407 ± 0.045	0.731^c^
CSFV	0.326 ± 0.068	0.328 ± 0.061	0.330 ± 0.067	0.666^c^
TIV	1.436 ± 0.143	1.416 ± 0.161	1.435 ± 0.134	0.512^d^
EFC index	0.611 ± 0.027	0.611 ± 0.025	0.610 ± 0.021	0.964^a^
SCD duration (years)	–	5.3 ± 8.0	–	–
SCD questionnaire	4.3 ± 2.2	14.8 ± 5.3	4.2 ± 2.2	<0.001^d^
MIG duration (years)	–	–	15.4 ± 9.4	–
MIG frequency (days/month)	–	–	6.4 ± 5.4	–
MIDAS			20.84 ± 14.03	
VAS			7.27 ± 2.06	
BDI score	7.8 ± 5.5	11.6 ± 8.0	6.1 ± 5.3	<0.001^d^
ISI total score (0–28)	9.4 ± 5.9	10.9 ± 6.2	6.8 ± 5.6	0.007^d^

All individuals with migraine or SCD, healthy controls were right-handed.

^a^Three-group analysis of variance test.

^b^Three-group chi-square test.

^c^Three-group analysis of covariance adjusted for age, sex, and total intracranial volume.

^d^Three-group analysis of covariance adjusted for age and sex.

BDI, beck depression inventory score; CSFV, cerebrospinal fluid volume; EFC, entropy focus criterion; GMV, gray matter volume; HC, healthy controls; ISI, insomnia severity index; MIDAS, Migraine Disability Assessment Questionnaire; MIG, migraine; SCD, subjective cognitive decline; TIV, total intracranial volume; WMV, white matter volume; VAS, visual analog scale.

#### 2.6.2. Analysis of SCNs of hippocampal subdivisions to identify network-level changes

Whole-brain voxel-wise statistical analyses were performed using SPM12. All voxel-wise anatomical findings were corrected for multiple comparisons using the cluster-extent thresholding approach with the updated version of related command-line tools (3dFWHMx and 3dClustSim, Analysis of Functional Neuroimages software, version 20.1.06; 10,000 Monte Carlo simulations with explicit GM mask). The significance level was set at a cluster-level family wise error (FWE) rate-corrected *p*-value of <0.05, which was equal to the combination threshold of an initial voxel-level *p*-value of <0.005 with a minimum cluster size of 235 voxels. For data reusability and transparency, all voxel-wise statistical maps without settled thresholds are available on the NeuroVault website.^4^ All analyses were performed on the voxel space and projected onto the brain surface for a more comprehensive visual presentation of the statistical results.

#### 2.6.3. Mapping of SCNs of hippocampal subdivisions in each study group

To determine anatomical regions that strongly co-varied with anterior or posterior hippocampal ROIs in GM volume, two separate voxel-wise general linear models were first constructed to correlate the mean GM volume of each seed ROI with GM volume measures across all GM voxels in each study group. The constructed model at each voxel *i* for a given seed ROI is specified as follows:


$$Y_{i}=intercept+\beta_{1}*V_{\text{seed}}+\beta_{2}*Age+\beta_{3}*Sex+\beta_{4}*$$

$$E\,d\,u\,c\,a\,t\,i\,o\,n+\beta_{5}*B\,D\,I+\beta_{6}*I\,S\,I+\beta_{7}*T\,I\,V+\varepsilon_{\text{i}},$$

where *Y* is the GM volume of voxel *i*; *V* is the mean GMV of the seed ROI of participants in a single study group; and nuisance variables are participants’ chronological age in years at the time of the scan, sex, education years, BDI score, ISI score, and TIV. The regression coefficients (β terms), intercept, and residual error (ε) were estimated using ordinary least squares. By assessing the significance level of β_1_, the potential SCN network for the corresponding seed ROIs in each study group was determined.

#### 2.6.4. Evaluating of the spatial similarity of anterior and posterior hippocampal SCN within each study group

To assess the spatial similarity of the SC pattern between the anterior and posterior hippocampus within each study group, unthresholded t-statistic maps were employed in conjunction with a spin permutation test. Initially, Pearson correlations were computed between the unthresholded t-statistic maps of SCN, derived from the anterior and posterior hippocampus seeds in each study group. Subsequently, the significance of spatial similarity was determined using a spatial spin permutation test approach, which involved 1,000 permutations. This method established a null distribution by comparing a target unthresholded t-statistic map with a permutated map generated by randomly rotating the spherical projections of the cortical surface while preserving the spatial relationships within the data (Alexander-Bloch et al., 2018). The relevant code for conducting the spin permutation test can be accessed via the following link.^5^

#### 2.6.5. Identifying distinct and shared network-level changes of hippocampal subdivisions

A two-step statistical approach was applied to identify distinct and shared network-level alterations of hippocampal subdivisions in individuals with SCD and migraine (Chou et al., 2021). First, two general linear interaction models were fitted to assess the case-control between-group differences (migraine vs. healthy control [HC]/SCD vs. HC) in SC strength for both anterior and posterior hippocampal ROIs. The constructed model at each voxel *i* for a given seed ROI is specified as follows:


$$Y_{i}=i\,n\,t\,e\,r\,c\,e\,p\,t+\beta_{1}*V_{\text{seed}}+\beta_{2}*G\,r\,o\,u\,p+\beta_{3}*\left(V_{s\,e\,e\,d}\times G\,r\,o\,u\,p\right)$$

$$+\beta_{4}*A\,g\,e+\beta_{5}*S\,e\,x+\beta_{6}*E\,d\,u\,c\,a\,t\,i\,o\,n+\beta_{7}*B\,D\,I+\beta_{8}*I\,S\,I$$

$$+\beta_{9}*T\,I\,V+\varepsilon_{i},$$

where *x* denotes the interaction between terms. By assessing the statistical significance of β_3_, case-control between-group differences in the SC strength between the predefined seed ROIs and the rest of the brain were determined. To further determine the shared network-level changes of hippocampal subdivisions between the SCD and migraine groups, a conjunction analysis was performed by searching the intersection of the voxel-wise FWE-corrected *p* maps obtained from the corresponding case-control between-group SCN analyses.

#### 2.6.6. Correlation of neuroanatomical data with clinical evaluations

For each anatomical cluster demonstrating a between-group difference (between HC and SCD or between HC and migraine) in SC, we calculated the corresponding SC integrity index for each individual and performed a series of partial Pearson’s correlation analyses between the SC integrity index and clinical evaluation in SCD and migraine groups (migraine duration and frequency, MIDAS, and VAS in the migraine group and SCD-Q score and SCD duration in the SCD group). Participants’ age, sex, education, ISI score, BDI score, and TIV were also used as nuisance variables in the correlational analyses. Of note, because the whole brain seed-to-voxel SCN analyses were conducted in a group-wise manner, a recently proposed deconstructed Pearson’s correlation coefficient approach (Eisenberg et al., 2015) was applied to obtain a single measurement to quantify the inter-regional SC integrities. This approach has also been utilized in studies investigating individual changes in structural connectivity integrity in patients with neurological and psychiatric disorders (Liu et al., 2020; Chou et al., 2021). Specifically, the group-wise structural covariance analysis was conducted by computing Pearson’s correlation coefficient between two distinct anatomical regions across participants. The Pearson’s correlation coefficient (*r*) between two brain regions (e.g., *X* and *Y* representing GM volume of two anatomical regions across study participants) can be expressed as the normalized inner product of their respective standard scores (z-scores):


$$r=\frac{1}{N-1}\,\sum_{i=1}^{N}\left(\frac{X_{i}-\bar{X}}{s_{X}}\right)\,\left(\frac{Y_{i}-\bar{Y}}{s_{Y}}\right)=\frac{1}{N-1}\,\sum_{i=1}^{N}z_{X_{i}}\,z_{Y_{i}},$$

where *N* denotes the number of participants per each study group; $\bar{X}$ and $\bar{Y}$ represent the mean value of *X* and *Y*, respectively; and s corresponds to the standard deviation of the study group. *z*_*X_i_*_ and *z*_*Y_i_*_ are equal to $\frac{X_{i}-\bar{X}}{s_{X}}$ and $\frac{Y_{i}-\bar{Y}}{s_{Y}}$, respectively, and *r* could be further regarded as the sum of a *partial_p* value of each individual of the corresponding study group; finally, *partial_p* of the individual i can be written as follows:


$$p\,a\,r\,t\,i\,a\,l\,\_\,p_{i}=z_{X_{i}}\,z_{Y_{i}}=\left(\frac{X_{i}-\bar{X}}{s_{X}}\right)\,\left(\frac{Y_{i}-\bar{Y}}{s_{Y}}\right)$$

Therefore, the *partial*_*p*_*i*_ could be used to indicate the contribution of each study participant *i* to the overall Pearson’s correlation coefficient of the corresponding study group and to serve as a surrogate measure of inter-regional structural coupling strength for that subject. The significance level was set at an uncorrected *p*-value of <0.05 for exploratory investigation.

## 3. Results

### 3.1. Demographic and clinical characteristics

Thirty-seven controls, 38 individuals with SCD, and 48 migraineurs were enrolled (Table 1). Sex (*p* = 0.7), age (*p* = 0.1), years of education (*p* = 0.08), global GM volume (*p* = 0.88), WM volume (*p* = 0.73), CSF volume (*p* = 0.67), TIV (*p* = 0.51), and EFC index (*p* = 0.96) did not differ among the groups. The mean duration of SCD and migraine was 5.3 ± 8.0 and 15.4 ± 9.4 years, respectively.

### 3.2. Spatial pattern of hippocampal subdivisions’ SCN

In controls, the anterior hippocampus showed significant structural coupling with the left hippocampus, right parahippocampal gyrus, bilateral lateral occipital cortex, left middle temporal gyrus, and left supramarginal gyrus (Figure 1B), whereas the posterior hippocampus demonstrated significant structural coupling with the bilateral hippocampus, bilateral lateral occipital cortex, and right supplementary motor cortex (Figure 1C). In individuals with SCD, the anterior hippocampus displayed significant structural coupling with the bilateral hippocampus, right posterior cingulate gyrus, and bilateral frontal pole (Figure 1B), whereas the posterior hippocampus showed significant structural coupling with the bilateral hippocampus, left frontal orbital cortex, right frontal pole, right middle temporal gyrus, right precentral gyrus (PreCG), and right posterior cingulate gyrus (Figure 1C). In migraineurs, the anterior hippocampus showed significant structural coupling with the left hippocampus, bilateral middle temporal gyrus, right cerebellum, and left lateral occipital cortex (Figure 1B). Meanwhile, the posterior hippocampus displayed significant structural coupling with the left hippocampus, right insular cortex, right anterior cingulate gyrus, right superior temporal gyrus, and left occipital pole (Figure 1C and Supplementary Table 1). Notably, the utilization of the spatial spin permutation test revealed significant spatial concordance between the anterior and posterior hippocampal SCNs within each study group (HC: *r* = 0.561, *p* = 0.001; SCD: *r* = 0.626, *p* = 0.001; and migraine: *r* = 0.677, *p* = 0.001).

### 3.3. Hippocampal subdivisions’ SCN integrity in SCD, migraine and controls

#### 3.3.1. SCN changes of the anterior hippocampus seed in SCD and migraine

**FIGURE 2:**
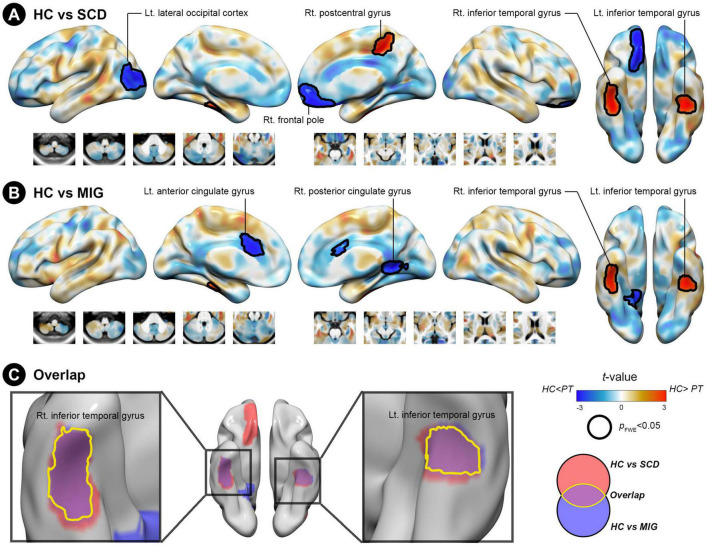
Structural covariance (SC) differences in the anterior hippocampus between the control and clinical groups (migraine and SCD). **(A)** Group-wise differences in SC between patients with migraine and controls. **(B)** Group-wise differences in SC between individuals with SCD and controls. Red/blue colors indicate decreased/increased SC in SCD and migraine compared with controls. Black outlines indicate significant regions at a corrected level for threshold *p*-values < 0.05. **(C)** Conjunction analyses showing SC alterations of the anterior hippocampus common to both clinical groups, located in the bilateral inferior temporal gyrus. FWE, family wise error; HC, healthy control; Lt, left; MIG, migraine; Rt, right; SC, structural covariance; SCD, subjective cognitive decline.

Compared with controls, individuals with SCD had decreased SC integrity in the right postcentral gyrus and bilateral inferior temporal gyrus (ITG), with the right frontal pole and left lateral occipital cortex showing increased SC integrity with the anterior hippocampus (Figure 2A and Table 2; FWE-corrected *p*-value < 0.05).

**TABLE 2:** Anatomical regions with significantly altered structural covariance of the anterior hippocampus in the migraine group, SCD group, and healthy controls.

MNI coordinates	Cluster size	Maximum *t*-value	Anatomical region	Integrity of structural covariance		
**x, y, z**				**HC**	**SCD**	**MIG**
**HC > SCD**						
41, −33, −21	712	4.15	Rt. inferior temporal gyrus	0.376*	-0.331	-0.262
−44, −33, −30	480	3.80	Lt. inferior temporal gyrus	0.295	-0.139	-0.163
17, −33, 50	395	3.43	Rt. postcentral gyrus	0.387*	0.331	0.352*
**HC < SCD**						
9, 50, −20	1300	4.71	Rt. frontal pole	0.012	0.562**	0.370*
−36, −90, 18	1168	4.22	Lt. lateral occipital cortex	-0.439*	0.112	-0.106
**HC > MIG**						
44, −16, −28	429	3.27	Rt. inferior temporal gyrus	0.400*	-0.315	-0.281
−41, −29, −28	281	3.25	Lt. inferior temporal gyrus	0.236	-0.211	-0.221
**HC < MIG**						
21, −42, 0	309	3.34	Rt. posterior cingulate gyrus	0.019	0.507*	0.163
−5, 20, 24	288	3.15	Lt. anterior cingulate gyrus	-0.119	0.051	0.281

Peak of group differences in integrity of structural covariance of anterior hippocampus with a threshold of FWE-corrected *p*-value < 0.05.

*p < 0.05. **p < 0.001.

HC, healthy controls; Lt, left; MIG, migraine; MNI, Montreal Neurological Institute; Rt, right; SCD, subjective cognitive decline.

Additionally, compared with controls, patients with migraine had regions of decreased SC integrity in the bilateral ITG, with the right posterior and left anterior cingulate gyri showing increased SC integrity with the anterior hippocampus (Figure 2B and Table 2; FWE-corrected *p*-value < 0.05).

The SCN alteration pattern was similar in the left and right anterior hippocampus in all groups (Supplementary Table 2). The conjunction analysis revealed that in both SCD and migraine groups, the anterior hippocampus showed decreased SC integrity with the right and left ITG (Figure 2C), whereas no region showed shared increased SC integrity with the anterior hippocampus.

#### 3.3.2. SCN alterations of the posterior hippocampus seed common to SCD and migraine

**FIGURE 3:**
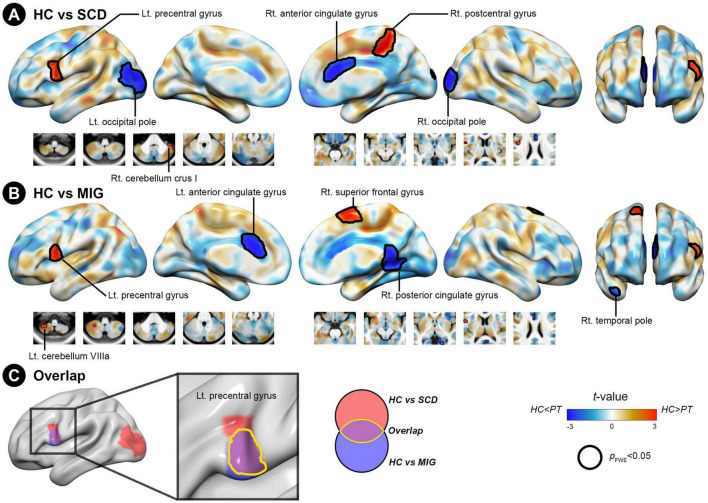
Structural covariance differences in the posterior hippocampus between the control and clinical groups (migraine and SCD). **(A)** Group-wise differences in SC between patients with migraine and controls. **(B)** Group-wise differences in SC between individuals with SCD and controls. Red/blue colors indicate decreased/increased SC in SCD and migraine compared with controls. Black outlines indicate significant regions at a corrected level for threshold *p*-values < 0.05. **(C)** Conjunction analyses showing SC alterations of the posterior hippocampus common to both clinical groups, located in the left precentral gyrus. FWE, family wise error; HC, healthy control; Lt, left; MIG, migraine; Rt, right; SC, structural covariance; SCD, subjective cognitive decline.

Compared with controls, individuals with SCD had regions of decreased SC integrity in the left PreCG, right postcentral gyrus, and cerebellum crus I, with the bilateral occipital pole and right anterior cingulate gyrus showing increased SC integrity with the posterior hippocampus (Figure 3A and Table 3; FWE-corrected *p*-value < 0.05). Moreover, compared with controls, migraineurs had regions of decreased SC integrity in the left PreCG and cerebellum VIIIa and right superior frontal gyrus, with the right posterior cingulate gyrus, right temporal pole, and left anterior cingulate gyrus showing increased SC integrity with the posterior hippocampus (Figure 3B and Table 3; FWE-corrected *p*-value < 0.05). SCN alteration was similar in the left and right posterior hippocampus in all groups (Supplementary Table 3). Conjunction analysis revealed that the posterior hippocampus showed decreased SC integrity with the left PreCG in both SCD and migraine (Figure 3C), whereas no region showed shared increased SC integrity with the posterior hippocampus.

**TABLE 3:** Anatomical regions with significantly altered structural covariance of the posterior hippocampus in the migraine group, SCD group, and healthy controls.

MNI coordinates	Cluster size	Maximum *t*-value	Anatomical region	Integrity of structural covariance		
**x, y, z**				**HC**	**SCD**	**MIG**
**HC > SCD**						
16, −33, 51	642	3.93	Rt. postcentral gyrus	0.352	0.265	0.291
−50, 2, 18	339	3.84	Lt. precentral gyrus	0.391*	0.380*	0.268
54, −54, −39	278	3.53	Rt. cerebellum crus I	0.288	-0.332	0.314*
**HC < SCD**						
23, −98, 15	419	4.50	Rt. occipital pole	-0.585**	0.343	0.224
−35, −92, 15	1289	4.12	Lt. occipital pole	-0.34	0.261	0.229
2, 29, 18	436	3.60	Rt. anterior cingulate gyrus	-0.023	0.436*	0.134
**HC > MIG**						
14, 11, 69	563	3.89	Rt. superior frontal gyrus	0.332	0.295	-0.003
−50, 3, 15	342	3.73	Lt. precentral gyrus	0.397*	0.419*	0.209
−26, −50, −60	602	3.20	Lt. cerebellum VIIIa	0.376*	0.115	0.135
**HC < MIG**						
35, 27, −35	333	4.18	Rt. temporal pole	-0.087	0.358*	0.299
20, −43, 1	614	3.82	Rt. posterior cingulate gyrus	-0.021	0.408*	0.498**
−2, 22, 25	409	3.34	Lt. anterior cingulate gyrus	-0.272	0.309	0.155

Peak of group differences in the integrity of structural covariance of the posterior hippocampus with a threshold of FWE-corrected *p*-value < 0.05.

*p < 0.05; **p < 0.001.

HC, healthy controls; Lt, left; MIG, migraine; MNI, Montreal Neurological Institute; Rt, right; SCD, subjective cognitive decline.

### 3.4. Potential clinical significance of inter-regional SCN changes in SCD

After excluding 3 cases with an exceptionally short duration of SCD and 4 cases with an exceptionally long SCD duration, a subset of 31 participants was selected from the initial group of 38 SCD cases. The exploratory partial Pearson’s correlation analysis performed on this refined subset revealed that the SC integrity between the right cerebellum crus I and posterior hippocampus correlated with SCD duration (*r* = 0.41, *p* = 0.04). In the migraine group, there was a trend toward a correlation between the MIDAS score and the integrity of the SC linking the right temporal pole and posterior hippocampus (*r* = −0.30, *p* = 0.06). Additionally, a similar trend was observed between the VAS score and the integrity of the SC in the same region (*r* = −0.30, *p* = 0.06).

## 4. Discussion

The present study revealed that individuals with SCD and migraine had altered SC integrity in the anterior and posterior hippocampus, specifically in the temporal, frontal, occipital, cingulate, precentral, and postcentral areas. Conjunction analyses identified shared SC alterations of the anterior hippocampus with the ITG and the posterior hippocampus with the PreCG in both SCD and migraine. The SC integrity of the posterior hippocampus–cerebellum axis demonstrated an association with SCD duration. These findings suggest that specific vulnerable regions may be involved in the pathophysiology of SCD and migraine.

Our results showed SCN alterations of the anterior hippocampus with the postcentral gyrus, ITG, frontal pole, and lateral occipital cortex in individuals with SCD. Prior research found that the anterior hippocampus displayed functional connectivity alternations with the postcentral gyrus, occipital lobe, anterior temporal lobe, and orbitofrontal and inferior frontal gyrus (Tang et al., 2020), partially in accordance with the present results. Additionally, a recent functional MRI study investigating the differential role of hippocampal subregions in memory specificity and generalization observed that the posterior hippocampus formed a network of lateral parietal cortices and occipital visual cortices (Frank et al., 2019). Furthermore, there is growing recognition of cerebellar–hippocampal interactions in the collaborative nature of cognitive processes. Moreover, recent studies demonstrated the clinical significance of cerebellar–hippocampal functional connectivity for temporal and spatial processing (Yu and Krook-Magnuson, 2015). Although the specific pathways mediating cerebellar–hippocampal interactions remain vague, these aforementioned findings concur with our results that individuals with SCD showed altered SC integrity of the posterior hippocampus in the PreCG, postcentral gyrus, cerebellum crus I, occipital pole, and anterior cingulate gyrus. Thus, individuals with SCD may present differential SC patterns along the anterior to posterior axis of the hippocampus. To the best of our knowledge, this study is the first to investigate the SC of hippocampal subregions in SCD. These specific vulnerable regions in the anterior and posterior hippocampus with SC alternations may be involved in SCD pathophysiology. Our findings potentially provide insights into future neuroimaging studies on SCD.

Moreover, our results demonstrated that individuals with SCD exhibited higher SC integrity in the anterior and posterior hippocampus, specifically in the frontal pole, lateral occipital cortex, occipital pole, and anterior cingulate gyrus. Additionally, one study utilizing resting-state functional MRI found that the SCD group had elevated amplitude of low-frequency fluctuations in the slow-4 band of the right lingual gyrus, which is located on the medial aspect of the occipital lobe, when compared to the HC group (Wang et al., 2021). Furthermore, a magnetoencephalographic study revealed that individuals with SCD displayed increased connectivity in the posterior cingulate cortex compared to healthy controls (Cheng et al., 2020). These findings suggest that heightened connectivity in individuals with SCD may serve as a compensatory mechanism during the early stages of memory impairments or as temporary adaptations to subjective memory complaints. However, a longitudinal study is necessary to track these SCD participants and validate the findings (Cheng et al., 2020).

Conversely, our results showed altered SC integrity in the ITG and posterior and anterior cingulate gyri of the anterior hippocampus, as well as in the PreCG, superior frontal gyrus, temporal pole, cerebellum, and posterior and anterior cingulate gyri of the posterior hippocampus in migraineurs. Chong et al. (2017) demonstrated enhanced SC between the hippocampus and cortico-limbic network regions, which are involved in pain processing in the frontal, temporal, and parietal lobes and the cerebellar WM in migraineurs. Our study corroborates these earlier findings, further supporting the notion that this enhanced connectivity may serve as a compensatory mechanism in response to maladaptive stress in individuals with migraines, as the hippocampus is known to be involved in pain-related attention (Zhu et al., 2021). However, anterior vs. posterior delineations of the hippocampus were not assessed. We identified differential SC within the hippocampal subregions of migraineurs, which were not reported previously. Our findings re-emphasize the pivotal role of the hippocampus in migraine pathophysiology and suggest the potential implications of differential SC patterns along the long axis of the hippocampus in migraine.

Using conjunction analyses, we examined network-level alterations of the hippocampal subdivisions to identify shared neuroanatomical substrates between SCD and migraine. These analyses identified shared SC alterations of the anterior hippocampus with the ITG and the posterior hippocampus with the PreCG in both SCD and migraine. ITG participation in cognitive processes and neuronal loss corroborates with early pathological findings in amnestic mild cognitive impairment (MCI) and Alzheimer’s disease (AD) (Scheff et al., 2011). Alternatively, changes in cortical surface area in ITG (Messina et al., 2013) and hippocampal volume have also been detected in migraineurs (Liu et al., 2018), whose SC between the hippocampi and corticolimbic regions is stronger (Chong et al., 2017). The hippocampus is a pivotal brain region for language processing (Piai et al., 2016), emotional brain networks (Zhu et al., 2019), and AD symptoms (Vyas et al., 2020), and it is connected to the ITG via the inferior longitudinal fasciculus (ILF) (Lin et al., 2020). ILF abnormalities play a role in visual processing and language comprehension deficits in patients with dementia (Shin et al., 2019). Moreover, ILF WM integrity changes in migraineurs (Chong and Schwedt, 2015). However, these previous studies did not explore the relationship between the ILF and functional differentiation along the long axis of the hippocampus. Notably, in this study, the anterior hippocampus showed altered SC integrity in the ITG in both SCD and migraine. Taken together, these findings, including our and previous findings, suggest that altered SC integrity is a potential neuroimaging signature for SCD and migraine.

The PreCG is involved in emotion compensation and regulation in MCI and AD (Li et al., 2009; Franzmeier et al., 2017). Moreover, recent studies showed that hippocampal connectivity with the PreCG was significantly correlated with chronic stress exposure and acute stress regulation in older adults with amnestic MCI (McDermott et al., 2019), and the PreCG had abnormal connectivity with the hippocampus in schizophrenia (Zarei, 2018). Additionally, the PreCG participates in pain anticipation, and altered functional connectivity of the hippocampus with the PreCG is detected in migraineurs (Liu et al., 2018). However, these studies did not consider the long-axis specialization of the hippocampus. Notably, in our study, the posterior hippocampus showed alternative SC integrity with the PreCG in both SCD and migraine. The shared SC pattern alterations of the anterior and posterior hippocampus with the ITG and PreCG may be potential network signatures for the underlying pathogenesis shared by SCD and migraine.

In this study, we also observed a positive association of SC integrity between the cerebellum crus I and posterior hippocampus with SCD duration. The cerebellum crus I contributes to working memory (Rothman and Mattson, 2010). Cerebellar GMV changes in MCI and early onset AD (Jacobs et al., 2018) and hippocampal GMV changes in SCD have been demonstrated (Peter et al., 2014). Therefore, SC integrity between the cerebellum and posterior hippocampus might be a potential imaging signature for individuals with SCD.

In the migraine group, we observed a tendency toward a correlation between the MIDAS score and the integrity of the SC linking the right temporal pole and posterior hippocampus. Additionally, a similar trend was observed between the VAS score and the integrity of the SC in the same region. Consistent with our findings, a previous study demonstrated significant region-to-region volume interactions associated with the severity of allodynia, including hippocampal volume with the inferior frontal gyrus, planum temporale, and amygdala (Chong et al., 2017). However, further investigations with a larger sample size are warranted to strengthen the validation of our present findings.

This study has several limitations. First, we used strict and well-characterized diagnoses for SCD and migraine, resulting in a modest sample size. Further large-sized studies are required to generalize our results. Second, we included only structural MRI scans, and comprehensive biomarker data were insufficient. Future studies could incorporate multimodal MRI and relevant biomarkers, such as β-amyloid and tau proteins. Despite these limitations, this study makes unique contributions. The use of a large-scale neuroanatomical network analysis afforded the opportunity to systematically characterize inter-regional coordination between distinct anatomical brain areas.

## 5. Conclusion

This study identified shared alterations in SC patterns of the anterior and posterior hippocampus with the ITG and PreCG in individuals with SCD and migraine. Additionally, we found that SC integrity between the cerebellum and posterior hippocampus was associated with SCD duration. Our findings shed new light on the underlying network-level mechanisms of SCD and migraine and help provide an objective imaging signature for the comorbidity of SCD and migraine.

## Data availability statement

The raw data supporting the conclusions of this article will be made available by the authors, without undue reservation.

## Ethics statement

The studies involving human participants were reviewed and approved by the Institutional Review Board of the Tri-Service General Hospital. The patients/participants provided their written informed consent to participate in this study.

## Author contributions

C-LT, K-HC, C-PL, and F-CY: conceptualization and writing – original draft. K-HC and P-LL: formal analysis and methodology. K-HC, C-PL, and F-CY: writing – review and editing. All authors contributed to the data curation, read, and approved the final version of the manuscript.

## Footnotes

1
https://github.com/rordenlab/dcm2niix

2
https://github.com/poldracklab/mriqc

3
http://www.neuro.uni-jena.de/cat/

4
https://neurovault.org/collections/10478/

5
https://github.com/netneurolab/neuromaps

## Supplementary material

Supplementary Figure 1 The correlation plots of SCD duration vs. the SC integrity between posterior hippocampus and right cerebellum crus I in SCD group. The data points has shown the residual value after adjusting for the confounding effects by a general linear model. SCD, subjective cognitive decline; SC, structural covariance.
