# Supplementary material for: Shared alterations in hippocampal structural covariance in subjective cognitive decline and migraine
Source: Front Aging Neurosci. 2023 Jun 20;15:1191991. doi: 10.3389/fnagi.2023.1191991 (PMC10318340; doi:10.3389/fnagi.2023.1191991)
Supplement: Supplementary file 1 [file Table_1.DOCX]

Supplementary Material

Shared alterations in hippocampal structural covariance in subjective cognitive decline and migraine

Chia-Lin Tsai^1^, Kun-Hsien Chou^2,3^, Pei-Lin Lee^3^, Chih-Sung Liang^4^, Chen-Yuan Kuo^5^, Yu-Kai Lin^1,6^, Guan-Yu Lin^1^, Yi-Chih Hsu^7^, Po-Kuan Yeh^4^, Fu-Chi Yang^1,6^*, Ching-Po Lin^2,3,8^*

*** Correspondence:**Fu-Chi Yang, MD, PhD

E-mail: [fuji-yang@yahoo.com.tw](mailto:fuji-yang@yahoo.com.tw)

Ching-Po Lin, PhD

E-mail: [cplin@ym.edu.tw](mailto:cplin@ym.edu.tw)

# Supplementary tables

**Supplementary Table 1.** Brain areas involved in the structural covariance network of hippocampus subdivisions.

| **MNI Coordinates** | | | **Cluster  size** | **Anatomical Region** | **Maximum t-score** |
| --- | --- | --- | --- | --- | --- |
| **x** | **y** | **z** |  |  |  |
| **Seed: anterior hippocampus** | | | |  |  |
| *HC* | | |  |  |  |
| -26 | -15 | -20 | 4803 | Left Hippocampus | 15.74 |
| 35 | -8 | -21 | 12724 | Right Parahippocampal Gyrus | 9.60 |
| -60 | -33 | -9 | 1875 | Left Middle Temporal Gyrus | 5.45 |
| 35 | -63 | 42 | 2896 | Right Lateral Occipital Cortex | 5.00 |
| -17 | -77 | 41 | 465 | Left Lateral Occipital Cortex | 4.94 |
| -44 | -48 | 50 | 1004 | Left Supramarginal Gyrus | 4.60 |
| *SCD* | | |  |  |  |
| 26 | -11 | -20 | 6139 | Right Hippocampus | 14.11 |
| -27 | -12 | -20 | 7259 | Left Hippocampus | 11.85 |
| 3 | -30 | 44 | 837 | Right Posterior Cingulate Gyrus | 5.55 |
| 14 | 53 | -20 | 346 | Right Frontal Pole | 4.88 |
| -33 | 47 | -17 | 539 | Left Frontal Pole | 4.26 |
| *MIG* | | |  |  |  |
| -27 | -14 | -19 | 15348 | Left Hippocampus | 19.31 |
| -60 | -12 | -17 | 1131 | Left Middle Temporal Gyrus | 5.35 |
| 66 | -26 | -5 | 654 | Right Middle Temporal Gyrus | 5.11 |
| 14 | -45 | -47 | 2598 | Right Cerebellum IX | 4.66 |
| 47 | 2 | -30 | 694 | Right Middle Temporal Gyrus | 4.43 |
| -24 | -72 | 24 | 441 | Left Lateral Occipital Cortex | 4.08 |
| -57 | -35 | 9 | 912 | Left Superior Temporal Gyrus | 3.91 |
| 14 | -6 | 44 | 985 | Right Supplementary Motor Cortex | 3.74 |
| 36 | 27 | 2 | 728 | Right Frontal Orbital Cortex | 3.48 |
| 54 | 8 | 21 | 285 | Right Precentral Gyrus | 3.25 |
| **Seed: posterior hippocampus** | | | |  |  |
| *HC* | | |  |  |  |
| -30 | -35 | -6 | 2844 | Left Hippocampus | 12.09 |
| 29 | -33 | -5 | 3459 | Right Hippocampus | 11.09 |
| 35 | -59 | 50 | 1360 | Right Lateral Occipital Cortex | 5.55 |
| 14 | -12 | 45 | 327 | Right Supplementary Motor Cortex | 4.71 |
| -42 | -60 | 47 | 656 | Left Lateral Occipital Cortex | 4.69 |
| 59 | -60 | 5 | 352 | Right Middle Temporal Gyrus | 3.79 |
| 2 | 32 | -8 | 438 | Right Subcallosal Cortex | 3.79 |
| 62 | 6 | 29 | 518 | Right Precentral Gyrus | 3.41 |
| -13 | -37 | -29 | 309 | Left Cerebellum I-IV | 3.32 |
| *SCD* | | |  |  |  |
| -27 | -32 | -5 | 5252 | Left Hippocampus | 10.43 |
| 29 | -33 | -8 | 7207 | Right Hippocampus | 10.29 |
| -14 | 21 | -27 | 2748 | Left Frontal Orbital Cortex | 4.83 |
| 10 | 49 | 47 | 273 | Right Frontal Pole | 4.51 |
| 69 | -18 | -5 | 287 | Right Middle Temporal Gyrus | 4.48 |
| 33 | -12 | 71 | 374 | Right Precentral Gyrus | 4.41 |
| 3 | -30 | 45 | 614 | Right Posterior Cingulate Gyrus | 4.16 |
| 36 | 41 | -12 | 890 | Right Frontal Pole | 4.06 |
| 0 | -56 | 23 | 593 | Right Precuneous Cortex | 3.94 |
| 5 | 12 | 24 | 411 | Right Anterior Cingulate Gyrus | 3.81 |
| 26 | 35 | 49 | 292 | Right Frontal Pole | 3.33 |
| *MIG* | | |  |  |  |
| -27 | -33 | -5 | 34218 | Left Hippocampus | 15.63 |
| 42 | 18 | -3 | 1076 | Right Insular Cortex | 5.63 |
| 9 | -9 | 41 | 876 | Right Anterior Cingulate Gyrus | 4.87 |
| 69 | -30 | 9 | 1810 | Right Superior Temporal Gyrus | 4.39 |
| -18 | -90 | 18 | 1319 | Left Occipital Pole | 4.23 |
| 17 | 58 | 22 | 458 | Right Frontal Pole | 3.99 |
| -41 | 38 | -11 | 851 | Left Frontal Pole | 3.70 |
| 6 | 27 | 36 | 318 | Right Paracingulate Gyrus | 3.67 |
| -7 | 43 | 31 | 273 | Left Paracingulate Gyrus | 3.67 |
| 53 | 5 | 20 | 552 | Right Precentral Gyrus | 3.65 |
| 51 | -60 | 18 | 490 | Right Lateral Occipital Cortex | 3.51 |
| -4 | 62 | -12 | 376 | Left Frontal Pole | 3.41 |

Abbreviations: HC, healthy controls; Lt, left; MIG, migraine; MNI, Montreal Neurological Institute; Rt, right; SCD, subjective cognitive decline.

**Supplementary Table 2. Anatomical regions with significantly altered structural covariance of the bilateral anterior hippocampus in migraine patients, insomnia patients, and healthy controls.**

| **MNI coordinate** | **Cluster size** | **Maximum  t-value** | **Anatomical region** | **Integrity of structural covariance** | | |
| --- | --- | --- | --- | --- | --- | --- |
| **x, y, z** |  |  |  | **HC** | **SCD** | **MIG** |
| *Left anterior hippocampus* | | | |  |  |  |
| ***HC > SCD*** | | | |  |  |  |
| 41, -33, -21 | 567 | 3.97 | Rt. temporal fusiform cortex | 0.244 | -0.342 | -0.227 |
| -45, -33, -30 | 369 | 3.66 | Lt. inferior temporal gyrus | 0.16 | -0.182 | -0.131 |
| 17, -33, 50 | 360 | 3.35 | Rt. postcentral gyrus | 0.398* | 0.286 | 0.311* |
| ***HC < SCD*** | | | |  |  |  |
| 9, 49, -20 | 1883 | 4.32 | Rt. frontal pole | 0.084 | 0.537* | 0.355* |
| -36, -92, 14 | 1166 | 4.15 | Lt. occipital pole | -0.384* | 0.126 | -0.153 |
| -3, 66, -17 | 276 | 3.52 | Lt. frontal pole | -0.08 | 0.207 | 0.350* |
| -33, -90, -18 | 396 | 3.29 | Lt. lateral occipital cortex | -0.117 | 0.442* | 0.15 |
| ***HC < MIG*** | | | |  |  |  |
| 23, -43, -1 | 534 | 3.46 | Rt. posterior cingulate gyrus | -0.07 | 0.471* | 0.222 |
| *Right anterior hippocampus* | | | |  |  |  |
| ***HC > SCD*** | | | |  |  |  |
| 41, -33, -21 | 792 | 4.25 | Rt. temporal fusiform cortex | 0.456* | -0.315 | -0.311* |
| -39, -30, -23 | 548 | 3.94 | Lt. temporal fusiform cortex | 0.388* | -0.107 | -0.216 |
| 15, -35, 39 | 352 | 3.56 | Rt. postcentral cingulate gyrus | 0.336 | 0.338 | 0.392* |
| ***HC < SCD*** | | | |  |  |  |
| 11, 50, -21 | 752 | 4.85 | Rt. frontal pole | -0.153 | 0.540* | 0.357* |
| -36, -90, 19 | 1098 | 4.28 | Lt. lateral occipital cortex | -0.462* | 0.108 | -0.052 |
| ***HC > MIG*** | | | |  |  |  |
| -41, -30, -28 | 417 | 3.68 | Lt. temporal fusiform cortex | 0.343 | -0.186 | -0.287 |
| 45, -15, -27 | 578 | 3.59 | Rt. inferior temporal gyrus | 0.463* | -0.322 | -0.330* |
| ***HC < MIG*** | | | |  |  |  |
| -3, 21, 24 | 388 | 3.24 | Lt. anterior cingulate gyrus | -0.139 | 0.071 | 0.261 |

Peak of group differences in integrity of structural covariance of bilateral anterior hippocampus with a threshold of FWE-corrected *p*-value < 0.05.

*p<0.05

**p<0.001

Abbreviations: HC, healthy controls; Lt, left; MIG, migraine; MNI, Montreal Neurological Institute; Rt, right; SCD, subjective cognitive decline.

**Supplementary Table 3. Anatomical regions with significantly altered structural covariance of the bilateral posterior hippocampus in migraine patients, insomnia patients, and healthy controls.**

| **MNI coordinate** | **Cluster size** | **Maximum  t-value** | **Anatomical region** | **Integrity of structural covariance** | | |
| --- | --- | --- | --- | --- | --- | --- |
| **x, y, z** |  |  |  | **HC** | **SCD** | **MIG** |
| *Left posterior hippocampus* | | | |  |  |  |
| ***HC > SCD*** | | | |  |  |  |
| -50, 2, 18 | 296 | 3.87 | Lt. precentral gyrus | 0.367* | 0.271 | 0.23 |
| 17, -33, 50 | 522 | 380 | Rt. postcentral gyrus | 0.289 | 0.235 | 0.245 |
| 11, 5, 57 | 317 | 3.79 | Rt. supplementary motor cortex | 0.363* | 0.127 | 0.113 |
| 53, -53, -38 | 293 | 3.52 | Rt. cerebellum crus I | 0.278 | -0.34 | 0.239 |
| ***HC < SCD*** | | | |  |  |  |
| 24, -98, 14 | 442 | 4.46 | Rt. occipital pole | -0.548* | 0.341 | 0.195 |
| -35, -92, 15 | 1643 | 4.25 | Lt. occipital pole | -0.299 | 0.337 | 0.246 |
| 12, 51, -8 | 345 | 3.54 | Rt. frontal medial cortex | 0.068 | 0.641** | 0.213 |
| 50, -66, -15 | 304 | 3.36 | Rt. lateral occipital cortex | -0.006 | 0.466* | 0.247 |
| 2, 29, 15 | 401 | 3.33 | Rt. anterior cingulate gyrus | -0.039 | 0.388* | 0.13 |
| ***HC > MIG*** | | | |  |  |  |
| 7, 6, 60 | 713 | 3.95 | Rt. supplementary motor cortex | 0.334 | 0.204 | -0.001 |
| -50, 5, 14 | 314 | 3.69 | Lt. precentral gyrus | 0.365* | 0.369* | 0.171 |
| -27, -51, -57 | 991 | 3.30 | Lt. cerebellum VIIIa | 0.372* | 0.159 | 0.06 |
| ***HC < MIG*** | | | |  |  |  |
| 35, 27, -35 | 393 | 4.45 | Rt. temporal pole | -0.162 | 0.511* | 0.298 |
| 17, -42, 3 | 611 | 4.04 | Rt. posterior cingulate gyrus | -0.084 | 0.417* | 0.510** |
| -3, 21, 24 | 476 | 3.41 | Lt. anterior cingulate gyrus | -0.276 | 0.274 | 0.199 |
| ***SCD > MIG*** | | | |  |  |  |
| 9, 54, -5 | 319 | 3.78 | Rt. paracingulate gyrus |  |  |  |
| *Right posterior hippocampus* | | | |  |  |  |
| ***HC > SCD*** | | | |  |  |  |
| 16, -33, 51 | 777 | 3.98 | Rt. postcentral gyrus | 0.395* | 0.24 | 0.324* |
| -38, -32, -23 | 282 | 3.71 | Lt. temporal fusiform cortex | 0.173 | 0.01 | -0.062 |
| -48, 2, 19 | 416 | 3.70 | Lt. precentral gyrus | 0.383* | 0.354* | 0.315* |
| ***HC < SCD*** | | | |  |  |  |
| 23, -98, 15 | 338 | 4.39 | Rt. occipital pole | -0.583** | 0.301 | 0.21 |
| -35, -92, 15 | 807 | 3.85 | Lt. occipital pole | -0.392* | 0.136 | 0.139 |
| 2, 29, 18 | 387 | 3.72 | Rt. anterior cingulate gyrus | -0.024 | 0.446* | 0.131 |
| ***HC > MIG*** | | | |  |  |  |
| -41, -65, 42 | 305 | 3.74 | Lt. lateral occipital cortex | 0.517* | -0.114 | -0.146 |
| 14, 11, 69 | 339 | 3.70 | Rt. superior frontal gyrus | 0.332 | 0.386* | 0.024 |
| -50, 3, 17 | 331 | 3.61 | Lt. precentral gyrus | 0.390* | 0.425* | 0.22 |
| 9, -20, 17 | 322 | 2.81 | Rt. thalamus | 0.572** | 0.335 | 0.164 |
| ***HC < MIG*** | | | |  |  |  |
| 14, -42, 5 | 413 | 3.29 | Rt. posterior cingulate gyrus | -0.005 | 0.311 | 0.451* |
| -2, 23, 26 | 290 | 3.16 | Lt. anterior cingulate gyrus | -0.274 | 0.297 | 0.093 |

Peak of group differences in integrity of structural covariance of bilateral PCG with a threshold of FWE-corrected *p*-value < 0.05.

*p<0.05

**p<0.001

Abbreviations: HC, healthy controls; Lt, left; MIG, migraine; MNI, Montreal Neurological Institute; Rt, right; SCD, subjective cognitive decline.
