# Supplementary figures and images for: Shared alterations in hippocampal structural covariance in subjective cognitive decline and migraine
Source: Front Aging Neurosci. 2023 Jun 20;15:1191991. doi: 10.3389/fnagi.2023.1191991 (PMC10318340; doi:10.3389/fnagi.2023.1191991)

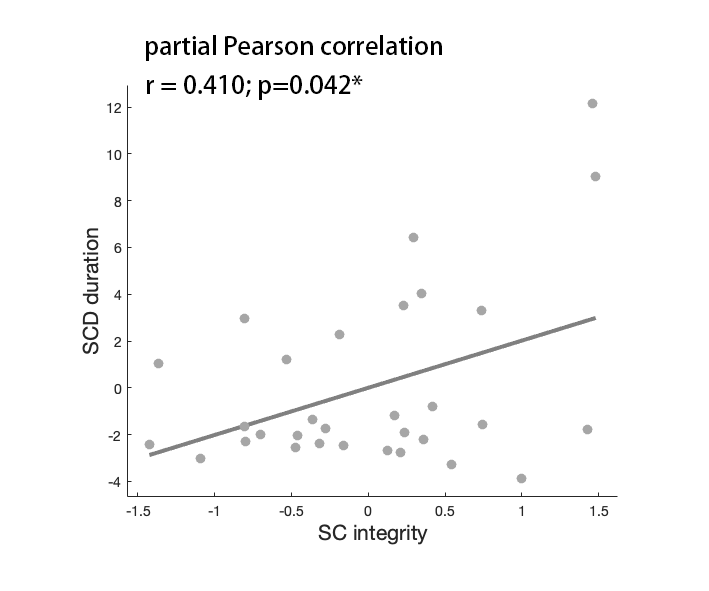

Supplement: Supplementary Figure 1 — The correlation plots of SCD duration vs. the SC integrity between posterior hippocampus and right cerebellum crus I in SCD group. The data points has shown the residual value after adjusting for the confounding effects by a general linear model. SCD, subjective cognitive decline; SC, structural covariance. [file Image_1.TIF]
